# Automated Calculation of Water‐equivalent Diameter (D_W_) Based on AAPM Task Group 220

**DOI:** 10.1120/jacmp.v17i4.6171

**Published:** 2016-07-08

**Authors:** Choirul Anam, Freddy Haryanto, Rena Widita, Idam Arif, Geoff Dougherty

**Affiliations:** ^1^ Department of Physics, Faculty of Mathematics and Sciences Diponegoro University Semarang Indonesia; ^2^ Department of Physics, Faculty of Mathematics and Sciences Bandung Institute of Technology Bandung Indonesia; ^3^ Applied Physics, California State University Channel Islands (CSUCI) California USA

**Keywords:** water‐equivalent diameter ($D_{W}$), size‐specific dose estimates (SSDE), volume CT dose index (${\text{CTDI}}_{\text{VOL}}$), patient dose

## Abstract

The purpose of this study is to accurately and effectively automate the calculation of the water‐equivalent diameter ($D_{W}$) from 3D CT images for estimating the size‐specific dose. $D_{W}$ is the metric that characterizes the patient size and attenuation. In this study, $D_{W}$ was calculated for standard CTDI phantoms and patient images. Two types of phantom were used, one representing the head with a diameter of 16 cm and the other representing the body with a diameter of 32 cm. Images of 63 patients were also taken, 32 who had undergone a CT head examination and 31 who had undergone a CT thorax examination. There are three main parts to our algorithm for automated $D_{W}$ calculation. The first part is to read 3D images and convert the CT data into Hounsfield units (HU). The second part is to find the contour of the phantoms or patients automatically. And the third part is to automate the calculation of $D_{W}$ based on the automated contouring for every slice ($D_{W,\text{all}}$). The results of this study show that the automated calculation of $D_{W}$ and the manual calculation are in good agreement for phantoms and patients. The differences between the automated calculation of $D_{W}$ and the manual calculation are less than 0.5%. The results of this study also show that the estimating of $D_{W,\text{all}}$ using $D_{W,n=1}$ (central slice along longitudinal axis) produces percentage differences of −0.92%±3.37% and 6.75%±1.92%, and estimating $D_{W,\text{all}}$ using $D_{W,n=9}$ produces percentage differences of 0.23%±0.16% and 0.87%±0.36%, for thorax and head examinations, respectively. From this study, the percentage differences between normalized size‐specific dose estimate for every slice (${}_{n}{\text{SSDE}}_{\text{all}}$) and ${}_{n}{\text{SSDE}}_{n=1}$ are 0.74%±2.82% and −4.35%±1.18% for thorax and head examinations, respectively; between ${}_{n}{\text{SSDE}}_{\text{all}}$ and ${}_{n}{\text{SSDE}}_{n=9}$ are 0.00%±0.46% and −0.60%±0.24% for thorax and head examinations, respectively.

PACS number(s): 87.57.Q‐, 87.57.uq‐

## I. INTRODUCTION

CT dose is an important concept in the medical community,^1^, ^2^, ^3^, ^4^ especially due to the growing use of computed tomography (CT) examinations^5^, ^6^ and concern over the relatively high dose from CT compared with other modalities.^7^, ^8^, ^9^ There is an ongoing need to estimate the radiation dose from CT examinations accurately. Currently, CT dose is characterized in terms of the CT Dose Index (CTDI) and dose‐length product (DLP).^10^, ^11^ CTDI values are widely used for quality assurance^(12)^ and accreditation purposes.^(13)^ However, CTDI was not intended to represent patient dose;^14^, ^15^ it was designed to approximate the average dose to the central slice of a cylindrical acrylic (polymethyl methaacrylate (PMMA)) phantom in a contiguous axial or helical examination.^(4)^

There are limitations to the CTDI values that are calculated using standard PMMA phantoms, which are typically 14 to 15 cm long and 16 cm in diameter to represent a patient's head or 32 cm in diameter to represent a patient's body. The first limitation is that the length of phantom is too short for measuring scattered radiation.^16^, ^17^, ^18^, ^19^ The second limitation is that the diameter of the phantom does not represent the variation in diameters among patients.^20^, ^21^, ^22^ For a given CT technique, the patient dose decreases as patient size increases, due to the increased attenuation of the incident X‐ray beam. The third limitation is that the phantom is homogenous, whereas a patient is composed of many materials with different attenuation properties.^23^, ^24^ For these reasons it has been recognized that CTDI represents the output radiation of the CT scanner rather than the patient dose.

Regarding the first limitation, CTDI is often measured using a 10 cm ionization chamber, which is considered too short because it excludes any contribution from radiation scattered beyond the relatively short range of integration along the Z‐direction, and it tends to undervalue the cumulative dose at the center position, as reported by many investigators.^16^, ^17^, ^18^, ^19^ To overcome this limitation, the American Association of Physicists in Medicine (AAPM) issued report TG‐111 which recommended using a sufficiently long (e.g., at least 45 cm) phantom to accommodate the scattered radiation.^(25)^ An alternative is to use a small‐volume ion chamber to directly measure the cumulative dose at any point by scanning the length of the phantom long enough to produce dose equilibrium in the center.^25^, ^26^

The second limitation has been evaluated by many authors who have reported that the dose to the patient depends on the size of patients.^20^, ^27^, ^28^ Huda et al.^(20)^ reported that the effective dose show an inverse correlation with increasing patient weight and size. The radiation dose was determined as a function of patient size (i.e., weight), ranging from young infants to overweight adults. However, AAPM provides methods that can be used to take into account the size of patient and, in 2011, introduced a new metric called the size‐specific dose estimates (SSDE).^(29)^In this report, patient size is estimated based on a geometrical parameter of the patient called the effective diameter (ED). Look‐up tables in the report provide conversion factors that can be applied to ${\text{CTDI}}_{\text{VOL}}$ to calculate SSDE. Conversion factors are based on four different measurements: anterior‐posterior (AP), lateral (LAT), AP+LAT, and effective diameter.^29^, ^30^

The effective diameter is a simple physical measure of patient size, and does not account for patient composition and attenuation properties.^23^, ^31^, ^32^ However, X‐ray attenuation is the fundamental physical parameter affecting the absorption of X‐rays and hence it determines the dose to the patient.^(33)^ This limitation was addressed by AAPM report TG‐220.^(34)^ In this report, AAPM used the concept of water‐equivalent diameter ($D_{W}$). It had previously been proposed by Huda et al.,^(20)^ Wang et al.,^(23)^ and Toth et al.^(35)^

The value of $D_{W}$ can be calculated either by a semiautomated (i.e., with some manual intervention) or by a fully automated method. The purpose of this study was to calculate the water‐equivalent diameter ($D_{W}$) from CT images using a fully automated method. The resulting estimate should be sufficiently accurate for computing the size‐specific dose estimate (SSDE). The study also investigated the difference between the water‐equivalent diameter using different numbers of slices, n (giving $D_{W,n}$), and the water‐equivalent diameter using all slices ($D_{W,\text{all}}$).

## II. MATERIALS AND METHODS

### A. The images of phantoms and patients

The objects for the $D_{W}$ calculations were standard CTDI phantoms (Leeds Test Objects (LTO) Medical Imaging Phantoms, Boroughbridge, UK) and patients. The phantoms consisted of two PMMA cylinders, one cylinder representing the head (16 cm diameter), and when nested inside the other representing the body (32 cm). The length of each cylinder was 14 cm. The head phantom was scanned by a CT scan Siemens SOMATOM Sensation 64 (Siemens Medical Solutions, Malvern, PA), installed at Dr. Karyadi Hospital, Semarang, Indonesia. The scanning parameters were: head spiral for adult study, slice thickness 0.3 cm, voltage 120 kVp, exposure 370 mAs, reconstruction diameter 18.7 cm, convolution kernel H20s. The body phantom was scanned by the same scanner with: abdomen routine for adult study, slice thickness 0.3 cm, voltage 120 kVp, exposure 200 mAs, reconstruction diameter 35.0 cm, and convolution kernel B30f.

There were 63 patients, 32 who underwent a CT head examination and 31 who underwent a CT thorax examination. The patients who underwent CT head examinations comprised 20 females and 12 males, with ages between 16 and 77 years. Those who underwent CT thorax examinations comprised 13 females and 18 males aged between 13 and 85 years.

The patients were scanned by a CT scan Siemens SOMATOM Emotion 6 at the Prof. Dr. Margono Hospital, Purwokerto, Indonesia. The scanning parameters for the head were: slice thickness 0.4 cm, voltage 130 kVp, tube current 167 mA, rotation time 1500 ms, reconstruction diameter 20.0 cm, number of slices 24, and convolution kernel H31s. The scanning parameters for the thorax were: slice thickness 1.0 cm, voltage 130 kVp, tube current 35–133 mA, rotation time 600 ms, reconstruction diameter 28.1 cm, number of slices 29–37, and convolution kernel B20s.

### B. The algorithm for automated $D_{W}$ calculation

In order to estimate $D_{W}$, AAPM suggested that the ROI be defined to include all of the patient, but no peripheral objects.^(34)^ A manually drawn ROI based on the patient contour could be used, but this technique requires more time and effort. Instead we implemented an automatically drawn contour around the patient.

**Figure 1:**
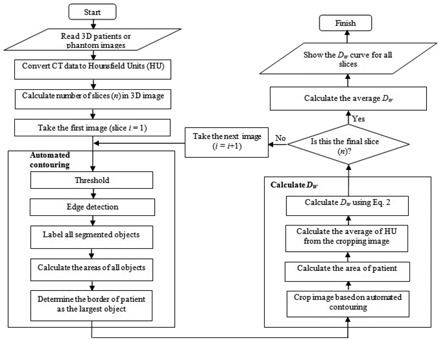
Flow chart for automated water‐equivalent diameter ($D_{W}$) calculation.

Figure 1 shows the flow chart for the automated $D_{W}$ calculation. There were three main parts to the algorithm. The first part is to read the 3D images and convert the CT data into Hounsfield units (HU). The second part is to contour the patients or phantoms automatically. And the third part is to automate the calculation of $D_{W}$ based on the automated contouring for every slice.

Some of the CT images were stored as CT data and some were stored in Hounsfield units (HU). A typical CT dataset ranges from 0 to 4095, although the real pixel values used only part of this range. Hounsfield units correspond to a scale from −1024 to +3071. The pixel value was displayed in the clinical application according to the average attenuation of the tissue on the Hounsfield scale. Water has an attenuation of 0 Hounsfield units (HU), air is −1000 HU, bone is typically +400 HU or greater, and metallic implants are usually 1000+ HU.

We applied a linear transformation to convert the CT data to Hounsfield units using Eq. (1), where *S* is the slope and *I* is the intercept. Normally, the value of slope and intercept are stored in the DICOM file itself. The tags are generally called the rescale slope and the rescale intercept, and typically have values of 1 and −1024, respectively. Tag (0028, 1052) is for the rescale intercept and tag (0028, 1053) is for the rescale slope.

(1) HU=CTdata×S+I

**Figure 2:**
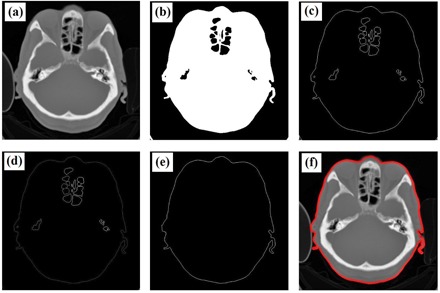
Steps for the automatic contouring process: (a) original image, (b) result of thresholding, (c) image after edge detection, (d) result of labeling, (e) patient's border image, and (f) result of autocontouring.

After converting the CT data to Hounsfield units, automated patient or phantom contouring was performed using an algorithm designed to produce accurate results with a relatively fast computation time. The algorithm uses a combination of basic segmentation techniques and specific information about the border of the patient body. The first step was thresholding, using a HU value of −200. This value was selected because the border between the patient and its surroundings is skin with HU values of approximately zero, while the surroundings outside the patient are air or other materials with HU values lower than −200. The thresholding produces binary images. However, thresholding alone was not be able to contour the patient completely because of the presence of other objects inside the patient with HU values lower than −200. To overcome this problem, we used edge detection to identify these objects and labeled them using their areas. The largest area identified was considered to be the border of the patient or phantom.^(32)^ The steps of this automated contouring are shown in Fig. 2.

After automated contouring, $D_{W}$ is calculated. The results of the automated contouring are used to crop the original images, and the area of the cropped image and the average HU value are calculated. $D_{W}$ is then calculated using Eq. (2), introduced by Wang et al.^(23)^ and adopted by AAPM 220:^(34)^

(2) $$D_{W}=2\sqrt{\left[\frac{1}{1000}\bar{HU{\left(x,y\right)}_{ROI}}+1\right]\frac{A_{ROI}}{\pi }}$$

where $A_{ROI}$ is the area of the patient after cropping and $\bar{HU{\left(x,y\right)}_{ROI}}$ is the average HU value of the patient or phantom. After the automated $D_{W}$ calculation is completed for one slice, it is continued for all the slices in the scan range. This gives the $D_{W}$ values for all slices, and the average $D_{W,\text{all}}$.

### C. Validation of $D_{W}$

The results of the automated $D_{W}$ calculation were compared to those calculated manually where the contouring was done freehand. Comparisons were made for both head and body phantoms, and for patient thorax and head. The automated and manual $D_{W}$ calculations for the patients were then correlated by regression.

In addition to the automated and manual calculations, the values of $D_{W}$ were also calculated theoretically for the phantoms. The phantoms were made from PMMA material with $\rho =1.19\;g/{\text{cm}}^{3}$, approximately 120 HU, 16 cm in diameter for head phantom and 32 cm in diameter for body phantom.

### D. Comparisons of $D_{W,n}$ to $D_{W,\text{all}}$

**Figure 3:**
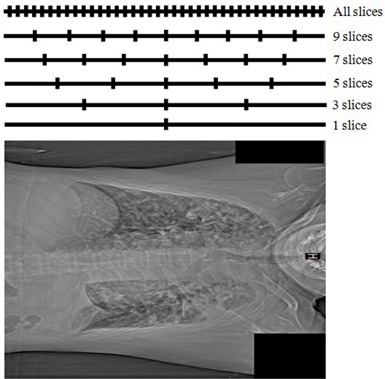
Slice positions for calculating $D_{W}$ along the longitudinal axis.

The calculation of water‐equivalent diameter using all slices ($D_{W,\text{all}}$) gives an accurate result. However, it is not practical clinically because it requires a long time for calculation, especially in the case of modern CT scanners that produce more than 500 images (i.e., slices) for every examination. We postulated that the value of $D_{W,\text{all}}$ could be estimated to an acceptable accuracy by using just a few (n) slices ($D_{W,n}$). In this study, we investigated the effect of using just n slices (n=1,3,5,7, and 9) to produce an estimate $D_{W,\text{all}}$. The position of every slice used for calculating $D_{W,n}$ and $D_{W,\text{all}}$ is indicated in Fig. 3.

The percentage difference (PD) between $D_{W,n}$ and $D_{W,\text{all}}$ was calculated using Eq. (3):

(3) $$PD=\left(\frac{D_{W,n}-D_{W,all}}{D_{W,all}}\right)\times 100\%$$

### E. Normalized ${\text{CTDI}}_{\text{VOL}}$ and SSDE calculation

The goal of the $D_{W}$ calculation is to estimate the size‐specific dose (SSDE). The SSDE value is determined by two main factors, namely the characteristic of the patient in terms of $D_{W}$ and the output dose of CT scanner in terms of the volume CT dose index (${\text{CTDI}}_{\text{VOL}}$).

The ${\text{CTDI}}_{\text{VOL}}$ values were calculated using ImPACT CT Patient Dosimetry Calculator version 1.0.1a. The exposure parameters (tube voltage, tube current, rotation time, slice thickness, pitch, and the type of phantom), manufacturer name and the type of scanner in each patient were put into the ImPACT CT in order to calculate the ${\text{CTDI}}_{\text{VOL}}$. Because the tube current for thorax examination spanned a wide range (35 mA to 133 mA), we calculated the normalized ${\text{CTDI}}_{\text{VOL}}$ (${}_{n}{\text{CTDI}}_{\text{VOL}}$) instead of ${\text{CTDI}}_{\text{VOL}}$ for both thorax and head examinations. The unit of ${}_{n}{\text{CTDI}}_{\text{VOL}}$ is mGy/100 mAs.

After determining the values of ${}_{n}{\text{CTDI}}_{\text{VOL}}$ and $D_{W}$, we calculated the value of normalized SSDE (${}_{n}\text{SSDE}$) using Eq. (4). The conversion factor $\left(k(D_{W})\right)$ from ${}_{n}{\text{CTDI}}_{\text{VOL}}$ to ${}_{n}\text{SSDE}$ is adapted from AAPM Task Group 220:

(4) $$n^{SSDE}{=}_{n}CTDI_{VOL}\times k(D_{W})$$

The value of $k(D_{W})$ depends on the type of phantom. For this study, there were two phantoms, namely the head CTDI phantom (16 cm in diameter) and the body CTDI phantom (32 cm in diameter).

## III. RESULTS

### A. $D_{W}$ for phantoms

**Figure 4:**
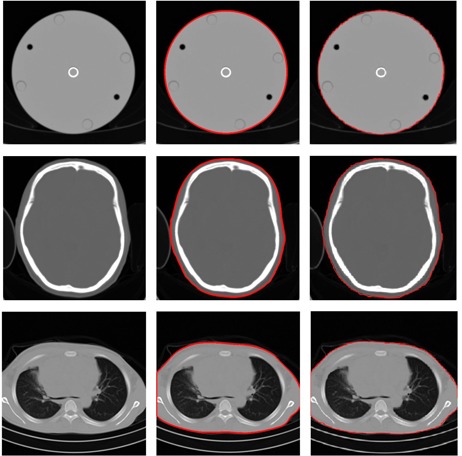
The first column shows the original images, the second column shows the results of autocontouring, and the third column shows the result of manual contouring. The first row shows images from the head phantom, the second row shows images from the patient head, and the third row shows images from the patient thorax.

**Figure 5:**
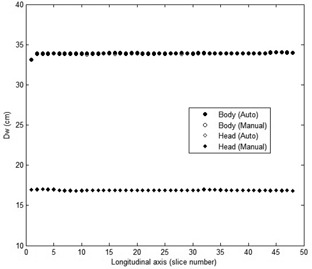
The $D_{W}$ values for the head and body phantoms based on automated and manual calculations along the longitudinal axis.

The results of contouring both automatically and manually are shown in Fig. 4. It can be seen visually that the results of automated contouring are similar to the manual contouring using free hand. The results of $D_{W}$ for the body and head phantoms using automated and manual calculations from the images and using theoretical calculations are shown in Table 1. The $D_{W}$ values for the body and head phantoms based on automated and manual calculations along the longitudinal axis are shown in Fig. 5.

The standard errors in the automated and manual methods are very small, indicating that the methods are precise. The theoretical result falls within the estimated errors (or just outside, in the case of the automated head phantom), indicating that the methods are accurate. The percentage differences the automated calculations to the manual calculations are less than 0.4%. The results indicate that the accuracy of automated calculation of $D_{W}$ is very good.

**Table 1:** The $D_{W}$ values for the head and body phantoms based on theoretical calculation, and automated and manual calculations from the CT image

	*Water‐equivalent Diameter* ($D_{W}$)		
*Phantoms*	*Theoretical Calculation*	*Manual Calculation*	*Automated Calculation*
Body	HU=120	33.92±0.13 cm	33.87±0.13 cm
	$\rho =1.19\;{g/\text{cm}}^{3}$		
	d=32 cm		
	$A=804.25\;{\text{cm}}^{2}$		
	$D_{W}=33.87\;\text{cm}$		
Head	HU=120	16.89±0.05 cm	16.87±0.04 cm
	$\rho =1.19\;{g/\text{cm}}^{3}$		
	d=16 cm		
	$A=201.06\;{\text{cm}}^{2}$		
	$D_{W}=16.94\;\text{cm}$		

### B. $D_{W}$ for patients

**Figure 6:**
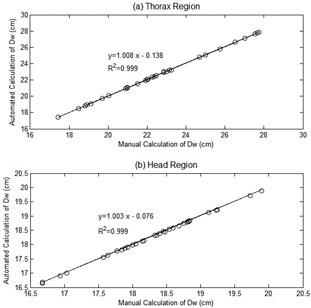
Graph of $D_{W}$ using automatic and manual calculations for (a) the thorax and (b) the patient head.

The automated and manual $D_{W}$ for the patient thorax and head are shown in Fig. 6. The resulting linear correlation coefficients between the automated and manual calculations gives $R^{2}=0.999$ for both the thorax and the head, and a slope of 1.008 for thorax and 1.003 for head. The manual values for $D_{W}$ are 22.48±2.88 cm and 18.29±0.79 cm for the thorax and the head, respectively. The automated values for $D_{W}$ are 22.54±2.90 cm and 18.28±0.79 cm for the thorax and the head, respectively. The percentage differences between the automated and manual calculations of $D_{W}$ are less than 0.5%, which is very small compared to the permitted tolerance, which is 10%.^(33)^ The results indicate that the automated calculations of $D_{W}$ from patient images are very close to the manual calculations.

### C. $D_{W}$ along the longitudinal axis

**Figure 7:**
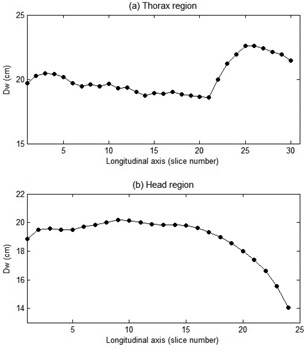
Profiles of $D_{W}$ values along longitudinal axis for (a) thorax and (b) head.

$D_{W}$ values along the longitudinal axis for the thorax and head for one of the patients are shown in Fig. 7. It shows that in the area of the lung, $D_{W}$ values are lower than the surroundings, because the main composition of the lung is air with HU value around −1000. Figure 7(b) shows that $D_{W}$ values in the head region are relatively flat, except in the area close to the apex of head.

AAPM TG‐220 recommended that water‐equivalent diameter be calculated as the average for every slice along the longitudinal axis ($D_{W,\text{all}}$). The average of $D_{W,\text{all}}$ for thorax and head examinations are listed in Table 2, which shows that the standard deviation of $D_{W,\text{all}}$ for head examinations is relatively small (±0.9 cm). On the other hand, the standard deviation of $D_{W,\text{all}}$ for thorax examination is relatively high (±2.6 cm). Table 2 also indicates the list of $D_{W,\text{all}}$ based on the gender. It can be seen that there is no significant difference of $D_{W,\text{all}}$ between male and female patients in the thorax region, but in the head region the $D_{W,\text{all}}$ value for female patients is slightly higher (about 1 cm) than for male patients.

Percentage differences between the average $D_{W,n}$ and $D_{W,\text{all}}$ are listed in Table 3. It shows that in the head region, the percentage difference between $D_{W,n=1}$ calculated from the central slice and the average $D_{W,\text{all}}$ is 6.75%±1.92%. It indicates that the value of $D_{W,n=1}$ calculated using the central slice is always greater than $D_{W,\text{all}}$. The percentage differences decrease when the number of slices (n) increases. The percentage difference for n=9 slices is 0.87%±0.36% or about 1%. On the other hand, in the thorax region, the average percentage difference between $D_{W,n=1}$ using the central slice and $D_{W,\text{all}}$ is relatively small (−0.92%), but there is a high standard deviation (±3.37%). The standard deviation decreases with increasing of number of slices used (n). At n=9, the standard deviation of percentage difference is very small (±0.16%).

**Table 2:** The $D_{W}$ values for thorax and head

	$D_{W}$ *(cm)*	
*Sex*	*Thorax*	*Head*
Male	22.87±2.44	17.86±0.62
Female	22.53±2.98	16.70±0.75
All	22.73±2.63	17.14±0.90

**Table 3:** Percentage differences of $D_{W,n}$ to the average $D_{W,all}$ for thorax and head

	*Percentage Differences of* $D_{W,n}$ *to the Average* $D_{W,\text{all}}$ *(%)*				
*Body Part*	*1 slice*	*3 slices*	*5 slices*	*7 slices*	*9 slices*
Thorax	−0.92±3.37	0.01±0.89	0.19±0.53	0.00±0.56	0.23±0.16
Head	6.75±1.92	4.11±1.11	3.03±0.75	2.47±0.62	0.87±0.36

### D. Normalized ${\text{CTDI}}_{\text{VOL}}$ and SSDE

**Figure 8:**
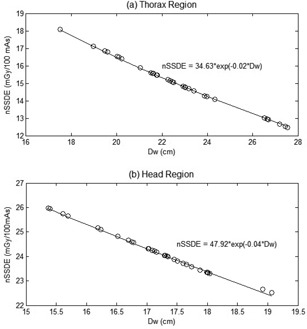
Normalized SSDE vs. $D_{W}$ for (a) thorax and (b) head.

The normalized ${\text{CTDI}}_{\text{VOL}}$ (${}_{n}{\text{CTDI}}_{\text{VOL}}$) values were 9.30 mGy/100 mAs and 25.10 mGy/100 mAs for thorax and head examination, respectively. The normalized SSDE (${}_{n}\text{SSDE}$) values based on a manual calculation of $D_{W}$ are 15.15±1.58 mGy/100 mAs and 23.15±0.70 mGy/100 mAs for thorax and head, respectively. The ${}_{n}\text{SSDE}$ values based on an automated calculation of $D_{W}$ are 15.12±1.59 mGy/100 mAs and 23.16±0.71 mGy/100 mAs for thorax and head, respectively. The percentage differences of ${}_{n}\text{SSDE}$ between the automated and manual calculations are less than 0.5%. As indicated in Fig. 8, the ${}_{n}\text{SSDE}$ values depend on $D_{W}$. It can be seen that the ${}_{n}\text{SSDE}$ decreases exponentially with increasing $D_{W}$.

The average ${}_{n}\text{SSDE}$ for thorax and head examinations is listed in Table 4. It shows that the standard deviation of ${}_{n}\text{SSDE}$ for head examination is smaller (±0.88 mGy/100 mAs) than for thorax examination (±1.44 mGy/100 mAs).

The percentage differences between ${}_{n}{\text{SSDE}}_{n}$ with n=1,3,5,7 and 9 slices to the ${}_{n}{\text{SSDE}}_{\text{all}}$ are shown in Table 5. The percentage differences between ${}_{n}{\text{SSDE}}_{n}$ with n=9 slices and the average ${}_{n}{\text{SSDE}}_{\text{all}}$ are very small (0.00%±0.46% and −0.60%±0.24% for the thorax and head examinations, respectively).

**Table 4:** The normalized SSDE values for thorax and head

	${}_{n}{\text{SSDE}}_{\text{all}}$ *(mGy/100 mAs)*	
*Sex*	*Thorax*	*Head*
Male	14.91±1.31	23.51±0.54
Female	15.13±1.65	24.65±0.76
All	15.00±1.44	24.22±0.88

**Table 5:** Percentage differences of ${}_{n}{\text{SSDE}}_{n}$ to the average of ${}_{n}{\text{SSDE}}_{\text{all}}$ for thorax and head

	*Percentage Differences of* ${}_{n}{\text{SSDE}}_{n}$ *to the Average of* ${}_{n}{\text{SSDE}}_{\text{all}}$ *%*				
*Body Part*	*1 slice*	*3 slices*	*5 slices*	*7 slices*	*9 slices*
Thorax	0.74±2.82	0.05±1.09	−0.01±0.74	−0.16±0.44	0.00+0.46
Head	−4.35±1.18	−2.05±0.72	−2.05±0.47	−1.69±0.40	−0.60±0.24

## IV. DISCUSSION

The aim of this study was to calculate the water‐equivalent diameter ($D_{W}$) automatically from axial CT images. A number of authors have reported the calculation of the patient diameter. Cristianson et al.^(28)^ calculated the effective diameter of patients automatically, but the calculation was performed on scout images using an adaptive threshold algorithm with a threshold set to 30% of the maximum pixel value. The limitation of that study, as reviewed by Pourjabbar et al.,^(30)^ is that the calculation of patient diameter only in the lateral or in the anterior‐posterior (AP) direction resulted in a high variability. In addition, the calculation of patient diameter from a scout image must be performed carefully. The patient centering must be set correctly, otherwise it can produce a magnified or minified image. Wang et al.^(23)^ showed that placing a 30 cm diameter water cylinder 5 cm and 9 cm closer to the X‐ray tube led to an overestimation of $D_{W}$ by 4.5% and 9.9%, respectively. In contrast, the calculation of the diameter for axial images requires no patient centering to attain high accuracy.

Patient contouring is very important in defining the patient. Care should be taken in defining the patient to include the entire patient, with only minimal portions of other attenuating materials such as the patient table. Including large amounts of the table would lead to an overestimate of $D_{W}$, particularly for small pediatric patients. Li et al.^(36)^ showed that the CT table can contribute up to 12% of the total attenuation for small objects, and Wang et al.^(23)^ reported that the patient table contributes up to 45.7% for a 7.7 cm wide phantom. Using our algorithm minimized the inclusion of parts outside the object (table).

Our method allows $D_{W}$ to be calculated automatically and with high accuracy. Accuracy is very important, since an overestimation of $D_{W}$ will lead to an underestimation of SSDE, and vice versa. Additionally, in our study, the percentage differences of the automated from manual $D_{W}$ calculations were less than 0.5%. The calculation using our algorithm is fast: using a standard notebook (Intel(R) Celeron CPU 1005M, 1.90 GHz, installed memory RAM 2.0 GB, and 32‐bit operating system), the automated calculation for each slice took less than 1 s.

Because patient dimension and attenuation can vary considerably along the longitudinal axis, the water‐equivalent diameter be calculated for every slice or for all positions along that axis ($D_{W,\text{all}}$). However, a previous study suggested that $D_{W}$ could be estimated from the central image in the scan range. Leng et al.^(24)^ reported that the average of $D_{W,\text{all}}$ correlated extremely well with a $D_{W,n=1}$ from a central image in the scan range. The important finding from this study is that the $D_{W,n=1}$ value of the central image approaches the value of average of $D_{W,\text{all}}$ only for thorax examinations. In the case of head examinations, however, the $D_{W,n=1}$ value of the central image is always greater than the average of $D_{W,\text{all}}$, with a percentage difference of 6.75%±1.92%. The $D_{W,n}$ values using n=9 slices produce a relatively small percentage difference (0.23%±0.16% and 0.87%±0.36% for thorax and head examinations, respectively), compared to $D_{W,\text{all}}$ for both thorax and head examinations.

There were several limitations in this study. First, the automated $D_{W}$ calculation was done to the patient images both for the full anatomy images and for the truncated anatomy images in the same way, without any correction for the latter images. In fact, the truncated anatomy images are missing part of the patient, and hence the calculation of $D_{W}$ is an underestimate, leading in turn to an overestimate of the SSDE. Truncation correction methods are very important to increase the accuracy of the $D_{W}$ measurement, especially in the thorax where the more peripheral distribution of attenuating tissue results in a proportionately larger truncation effect than in the more uniformly distributed abdominal tissue.^(37)^ Second, this algorithm was only applied to images that were taken using a fixed tube current (FTC). Nevertheless, the automated contouring achieved good precision and accuracy for all slices, even though they had different noise levels.

## V. CONCLUSIONS

We have proposed and tested a new algorithm for the automated calculation of water‐equivalent diameter ($D_{W}$). The percentage differences of the automated results from the manual $D_{W}$ calculations were less than 0.5%, which is promising for the implementation of this method in the clinical environment. We found that the average value of water‐equivalent diameter from all slices ($D_{W,\text{all}}$) could be estimated accurately using slices only, distributed along the longitudinal axis ($D_{W,n}$). Using nine slices, the percentage difference between $D_{W,n=9}$ and $D_{W,\text{all}}$ was about 1%.

## ACKNOWLEDGMENTS

The authors are grateful for the funding of this work by the Research and Innovation Program, Bandung Institute of Technology (ITB), No. 237h/I1.C01/PL/2015 and No. 006n/I1.C01/ PL/2016. The authors would like to thank Mr. Masdi from Prof. Dr. Margono Hospital, Purwokerto, Indonesia.

## COPYRIGHT

This work is licensed under a Creative Commons Attribution 3.0 Unported License.
